# Comparison of Pressure Sensing Properties of Carbon Nanotubes and Carbon Black Polymer Composites

**DOI:** 10.3390/ma15031213

**Published:** 2022-02-06

**Authors:** Jongchan Yoo, Dong-Young Kim, Hyunwoo Kim, Oh-Nyoung Hur, Sung-Hoon Park

**Affiliations:** Department of Mechanical Engineering, Soongsil University, 369 Sangdo-ro, Dongjak-gu, Seoul 06978, Korea; dbwhdcks39@gmail.com (J.Y.); qnfdydlf013@gmail.com (D.-Y.K.); kimhw3@gmail.com (H.K.); ohnyung324@soongsil.ac.kr (O.-N.H.)

**Keywords:** carbon black, carbon nanotube, piezoresistive effect, pressure sensor, polymer composite

## Abstract

Polymer composites containing conductive fillers that utilize the piezoresistive effect can be employed in flexible pressure sensors. Depending on the filler used, different characteristics of a pressure sensor such as repeatability, sensitivity, and hysteresis can be determined. To confirm the variation of the pressure sensing tendency in accordance with the dimensions of the filler, carbon black (CB) and carbon nanotubes (CNTs) were used as representative 0-dimension and 1-dimension conductive fillers, respectively. The piezoresistive effect was exploited to analyze the process of resistance change according to pressure using CB/PDMS (polydimethylsiloxane) and CNT/PDMS composites. The electrical characteristics observed for each filler were confirmed to be in accordance with its content. The pressure sensitivity of each composite was optimized, and the pressure-sensing mechanism that explains the difference in sensitivity is presented. Through repeated compression experiments, the hysteresis and repeatability of the pressure-sensing properties were examined.

## 1. Introduction

Over the past decade, promising applications of pressure sensors such as motion detection [1,2], health monitoring [3,4] and tactile-sensitive skin [5,6] have received significant attention. This has driven demand for wearable and flexible pressure sensors. Consequently, various studies on pressure sensors made of conductive polymer composites (CPCs) have been conducted as alternatives to conventional metallic sensors possessing low sensitivity and flexibility [7,8]. Recently, active research has been focused on the manufacture of CPCs using carbon nanomaterials as conductive fillers [9,10].

A CPC study reported that, according to the principle of sensing, pressure sensors are divided into different categories such as the sensors based on piezoelectric effect [11,12], capacitive effect [13,14], and piezoresistive effect [15,16]. CPC pressure sensors based on the piezoresistive effect are generally used because of their easy manufacturing and high sensitivity [17,18]. The piezoresistive effect is defined as the change in electrical resistance when subjected to mechanical strain [19]. The electrical properties of CPC pressure sensors with piezoresistive effects are significantly affected by the shape, concentration, and dispersion of the filler [20,21,22,23,24].

However, the mechanism of pressure-sensing behavior in a pressure field remains unclear. Therefore, a comparative study on carbon fillers is useful for understanding the pressure sensing mechanism, and this research will enhance their applicability in pressure sensors. Furthermore, detailed studies on the effect of filler dimensions are still inadequate, providing important directions for future research on compressive sensors.

In this study, carbon nanotubes (CNTs) and carbon black (CB), which are representative conductive carbon nanofillers, were used to compare the pressure-sensing properties of different geometric structures. The three-roll milling method was used to prepare CNT/PDMS (polydimethylsiloxane) and CB/PDMS CPCs, in which the fillers were evenly dispersed. The morphology, dispersion, and percolation behavior were analyzed first. To examine the differences in the reproducibility and sensitivity of the two types of CPCs, the change in resistance with pressing was recorded. Finally, the tendency and mechanism of pressure sensing was investigated through a hysteresis comparison according to the internal structure and repeated experiments after 50 pressing cycles.

## 2. Materials and Methods

### 2.1. Materials

CB and CNTs were used as the conductive fillers. CB with a 0-dimensional diameter of 34 nm and a true density of 1.7 g/m^3^ was purchased from Mitsubishi Chemical Corporation (Tokyo, Japan). CNTs were purchased from KB-Element (Gyeonggi-do, Korea) and their average outer diameter, bundle length, and a true density were 5 nm, 10–20 μm, and 1.4 g/m^3^, respectively. Polydimethylsiloxane (PDMS; Sylgard 184, Dow Corning, Midland, Ml, USA) was used as the base polymer matrix.

### 2.2. Fabrication of CB/PDMS and CNT/PDMS Composite

The fabrication of the CB/PDMS and CNT/PDMS composites with different filler concentrations was carried out using a paste mixer (Daehwa, Seoul, Korea) and a three-roll milling machine (Intech, Gyeonggi-do, Korea) [25]. First, the PDMS was prepared with a mass ratio of the curing agent to the base of 1:10. Next, CNTs and CB were premixed with PDMS using a paste mixer for 30 s at 500 rpm and then for 1 min at 1500 rpm. The CNTs and CB were then evenly dispersed in the composites using a three-roll milling machine for 5 min. The mixture was then pressed and cured using a hot press (Qmesys Inc., Gyeonggi-do, Korea) at 180 °C for 1 h and 13 MPa to obtain a 500 μm thick flat film.

### 2.3. Characterization and Test Conditions

The CPCs were fractured in liquid nitrogen to confirm the morphology and dispersion of CNTs and CB, respectively, in the polymer matrix. The cross-sectional surface of the fractured CPCs was observed using a Gemini SEM 300 instrument (Zeiss, Land Baden-Württemberg, Germany). The acceleration voltage of the equipment was maintained at 5 kV.

Five different CPC samples were prepared and tested for each composition to measure the electrical properties and penetration threshold of CPCs. The specimen dimensions were 50 × 5 × 0.5 mm^3^. UV etching and silver paste were used to reduce the contact resistance and measure the electrical properties more precisely. First, the specimen was surface treated for 300 s in a UV ozone chamber machine (JSE Co., Seoul, Korea) to improve contact with the electrode. Next, silver paste (Protavic, Levallois-Perret, France) was applied as an electrode at both ends of the specimen. After curing the electrodes at 120 °C for 1 h, the resistance was measured using the two-point method with a Keithley DMM 7510 multimeter (Keithley, Cleveland, OH, USA).

**Figure 1:**
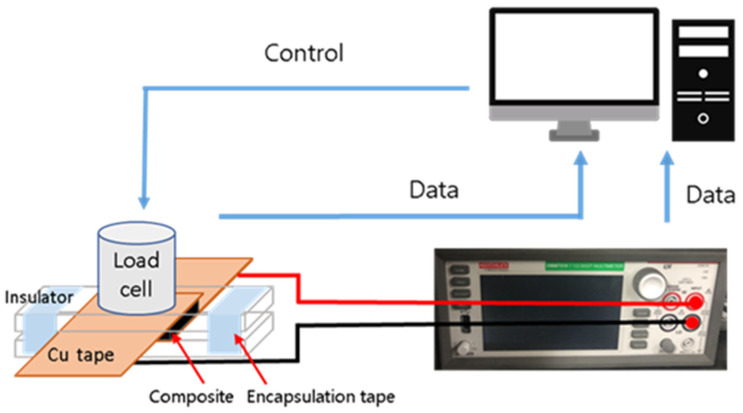
Scheme of the pressure sensing experimental setup.

To measure the change in electrical properties with pressure on the CPCs, two Cu tapes as electrodes attached to the substrate were placed between the samples. However, it is difficult to clearly observe the piezoresistive effect due to the contact resistance between the CPC and the electrode [26,27]. This problem was resolved in this study by placing a 500 g weight and encapsulating the experimental setup with tape so that the electrode and CPC were in tight contact. To measure the pressure-sensing behavior, the resistance was recorded with a Keithley DMM while simultaneously applying pressure using a custom press machine with a strain gauge (NAMIL, Incheon, Korea). The load cell compressed the CPC in the pressure range of 0–180 kPa, and the speed was set to 1 mm/min. Hysteresis characteristics were identified through 30 cycles of load-unload experiments in a pressure range of 0–40 kPa and a 30-s delay for each process. Subsequently, the loading-unloading experiment was performed in a pressure range of 0–40 kPa without delay for 3000 s. Figure 1 schematically presents pressure sensing experimental setup.

## 3. Results and Discussion

### 3.1. Morphology Analysis

**Figure 2:**
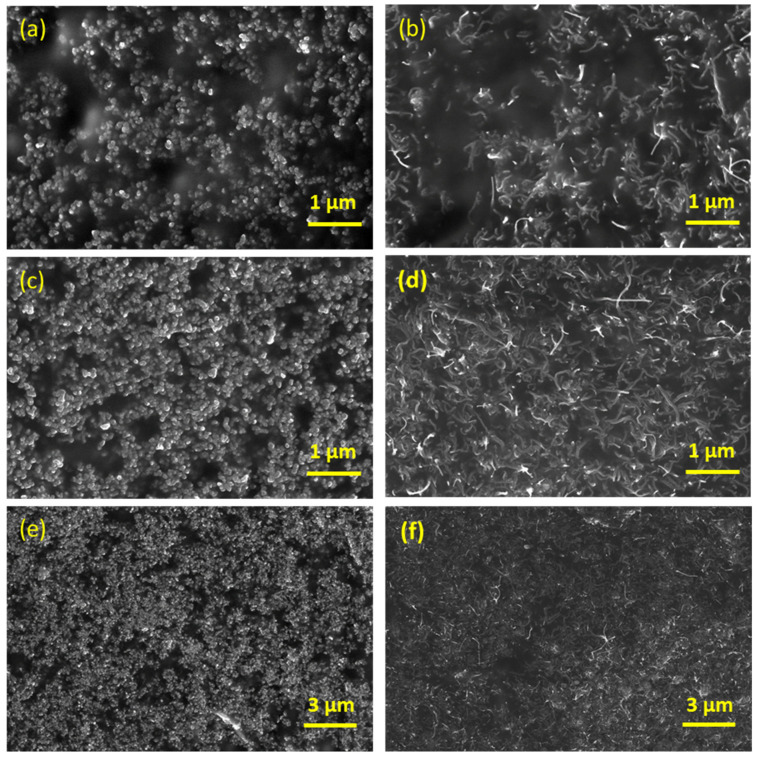
SEM images of composites at high resolution: (**a**) CB/PDMS 5 wt.%, (**b**) CNT/PDMS 5 wt.%, (**c**) CB/PDMS 10 wt.%, (**d**) CNT/PDMS 10 wt.%; at low resolution: (**e**) CB/PDMS 10 wt.%, (**f**) CNT/PDMS 10 wt.%.

Figure 2 shows the SEM images of the fractured surfaces of the low-and high-content carbon black and carbon nanotube composites (CB/PDMS and CNT/PDMS, respectively) synthesized in this study. Figure 2a,b show cross-sectional SEM images of the composite materials prepared with 5 wt.% of CB and CNTs. Figure 2c–f show cross-section of 10 wt.% CB and CNTs composites at low (c,d) and high resolution (e,f). The SEM images depict the typical shape of the nanofillers and the differences according to the dispersion and content of the nanofillers. Therefore, it can be observed from Figure 2 that CB has a typical 0-dimensional spherical shape, and CNT has a typical 1-dimensional shape with a long aspect ratio, and all fillers are well dispersed in polymer matrix regardless of the contents.

### 3.2. Electrical Conductivity and Percolation Threshold

**Figure 3:**
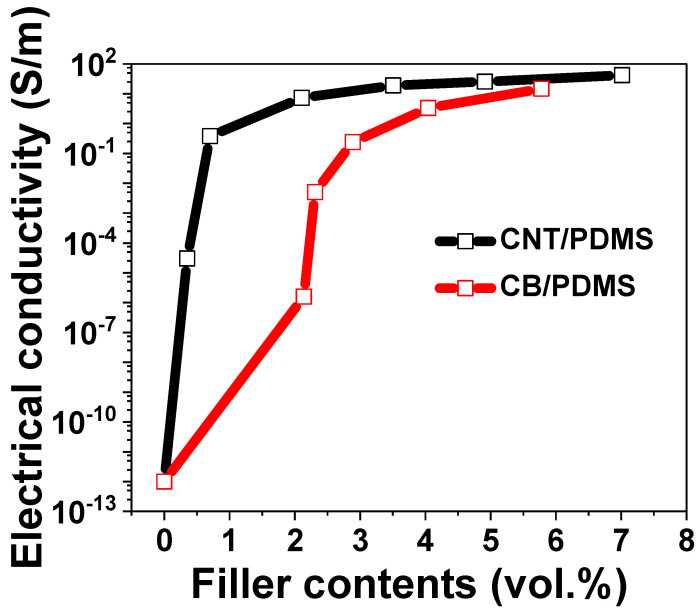
The electrical conductivity of CB/PDMS and CNT/PDMS composites as a function of filler contents (vol%).

To determine the electrical behavior of CB/PDMS and CNT/PDMS in a pressure field, the percolation behavior was investigated. The electrical properties of CPCs extrinsically depend on the shape, type, and content of the filler. Figure 3 shows the effect of filler content on the electrical conductivity and percolation threshold of CB/PDMS and CNT/PDMS. The electrical conductivity of the CPCs was determined according to the following equation:

(1) $$\sigma_{conductivity}=\frac{l}{RA}$$

where $\sigma_{conductivity}\;$is the electrical conductivity, l is the distance between the electrodes, R is the electrical resistance of the sample and A is the cross-sectional area.

CNTs with large aspect ratios can easily form electrical networks among them. Therefore, a sudden increase in electrical conductivity is observed at lower content values for CNT/PDMS than for CB/PDMS owing to more electrical pathways, even when placed in a smaller volume [28,29]. Owing to the percolation phenomenon, insulating polymers become electrically conductive when the filler content exceeds a certain threshold [30]. According to the percolation theory, the relationship between the conductivity of the composite and the filler content is defined as follows:

(2) $$\sigma_{c}\propto \sigma_{0}{\left(V_{f}-V_{fc}\right)}^{t}\;where\;V_{f}>V_{fc}$$

where $\sigma_{c}$ denotes the electrical conductivity of the composite, $\sigma_{0}\;$is a constant, $V_{f}$ denotes the volume fraction of the filler, $V_{fc}$ denotes the volume fraction of the fillers at the percolation threshold, and t is the critical index. In the present study, $V_{fc}$ and *t* calculated using Equation (2) were 0.35 vol.% and 0.92 for CNT/PDMS and 2.13 vol.% and 3.28 for CB/PDMS, respectively. The obtained results ($V_{fc}$ and *t*) follow the same trends as those shown in previous studies [20,31,32]. This study observed a lower percolation threshold for CNT/PDMS as compared to that for CB/PDMS. Consequently, it was confirmed that CPCs can be manufactured by adding a small amount of CNTs as compared to CB.

### 3.3. Pressure Sensing Properties

#### 3.3.1. Sensitivity

**Figure 4:**
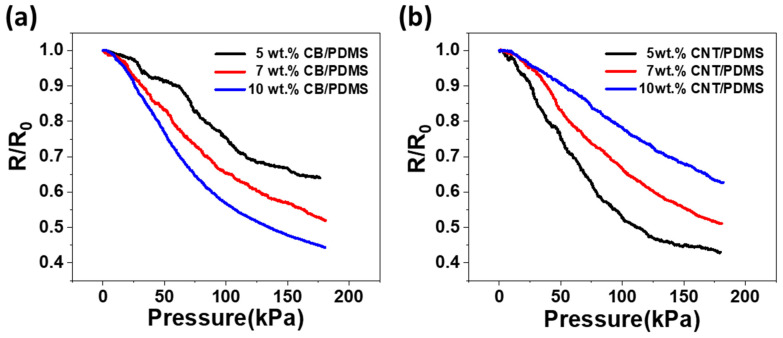
Normalized change in resistance (R/R_0_) versus pressure with varying contents of (**a**) CB and (**b**) CNTs in the composites.

Pressure measurements were performed with different contents to compare the piezoresistive behaviors of the CB/PDMS and CNT/PDMS composites. Figure 4 shows the variation of relative resistance (R/R_0_) of the CPCs with 5, 7, and 10 wt.% of CB (Figure 4a) or CNTs (Figure 4b) with respect to the pressure up to 180 kPa, where R_0_ is the initial resistance. It is difficult to stably detect the resistance of composites whose contents are close to the percolation threshold because of their high electrical resistance with current spikes (noise) [33,34]. Therefore, for this pressure test, relatively high filler contents (above the percolation threshold) were selected to obtain a stable resistance signal.

Pressing the CPCs decreases the resistance which can be explained by two reasons. One is the complete contact with the electrode on pressing, and the other is the piezoresistive effect. Rajarajan et al. [26] reported that when pressure is first applied under no load, the resistance changes rapidly, which is mainly due to full contact with the electrode and is called the nominal pressure. For the subsequent change in the resistance with pressure, the main factor responsible is the piezoresistive effect. In this paper, to more reliably identify the piezoresistivity of CPCs, a pressure of 21.8 kPa was applied to the experimental setup and encapsulated with tape. Then, the load cell was pressed onto the enclosed experimental setup.

During the piezoresistive effect, the main reason for the resistance change is that the distance between adjacent fillers is reduced and a conduction path is created [33]. Thus, in this study, the pressure caused a decrease in the resistance of all the fabricated CPCs. In Figure 4a, it can be seen that the higher the filler content, the higher is the sensitivity to pressure in the CPCs-containing CB. In this experiment, the resistance change rate of the 5 wt.% CB/PDMS composite is the largest, and the resistance change rate of 10 wt.% CB/PDMS composite is the smallest. This is because when a low CB content is used, the unstably connected conductive paths can be destroyed. Increasing the amount of CB in the matrix increased the electrical network and made it more reliable. Therefore, when a high CB content is used, more conductive paths are created, even if they are destroyed by pressure. On the other hand, as shown in Figure 4b, it can be inferred that the lower the filler content in CPCs-containing CNTs, the higher is the pressure sensitivity. In this test, the resistance change rate of 10 wt.% CNT/PDMS composite is the largest, and the resistance change rate of 5 wt.% CNT/PDMS composite is the smallest. This shows the opposite tendency to that of the composite containing CB. Unlike CB, CNTs, which are one-dimensional materials, are more likely to produce contact points, allowing stable electrical paths to be formed, even at low concentrations. However, when the CNTs content is high, it is difficult to create a new electrical network owing to sufficient contact, resulting in a lower sensitivity to pressure than when using a low CNTs content. In subsequent experiments, 10 wt.% CB/PDMS and 5 wt.% CNT/PDMS, which have the highest sensitivity to pressure for each filler, were studied and compared.

#### 3.3.2. Piezoresistive Effect under Cyclic Pressure

In CPCs based on viscoelastic polymers, a hysteresis phenomenon is observed, in which the electrical conductivity does not return to its initial state even when the external force is removed [35,36]. In CPC pressure sensors, hysteresis causes instability under pressure, resulting in different loading and unloading response curves. However, this phenomenon has been reduced in the initial repeated pressure cycle through relocation of the fillers inside the CPCs, ensuring stability inside the CPCs [37]. This rearrangement depends significantly on the morphology of the filler [38]. In the present study, a 30 cycle load-unload experiment was performed on CB/PDMS and CNT/PDMS composites to investigate the hysteresis variation according to the filler.

**Figure 5:**
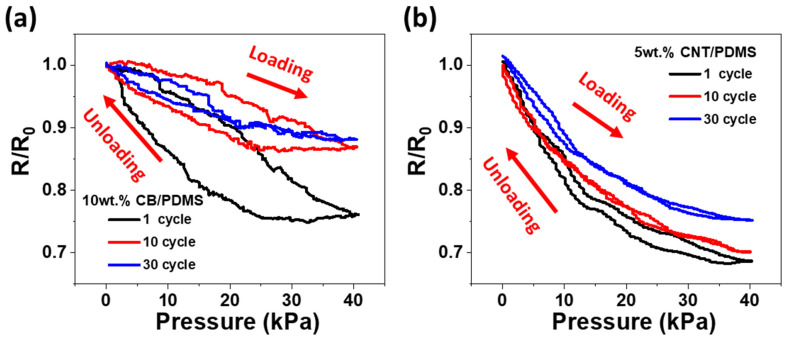
Normalized change in resistance (R/R_0_) with cyclic pressure deformation of 40 kPa for (**a**) 10 wt.% CB/PDMS and (**b**) 5 wt.% CNT/PDMS composites.

Figure 5 displays the change in the relative resistance of the CPCs under the loading and unloading cycle test at 40 kPa. The 10 wt.% CB/PDMS and 5 wt.% CNT/PDMS composites with the highest sensitivity were tested and the curves for 1, 10, and 30 cycles are shown in Figure 5a,b, respectively. As shown in Figure 5a, 10 wt.% CB/PDMS composite shows significantly different loading and unloading response curves in the initial cycle, which means that hysteresis is large. In contrast, as shown in Figure 5b, 5 wt.% CNT/PDMS composite has little hysteresis in the initial cycle. As the number of cycles increased, a difference was observed in the rate of change in the resistance with pressure. As can be observed from Figure 5a, the resistance of the CB/PDMS composite in first cycle dropped to 75% and to 88% at 30th cycle, resulting in a net 13% reduction in the resistance change at 30th cycle with respect to the first cycle. On the other hand, as shown in Figure 5b, the resistance of the CNT/PDMS composite in first cycle decreased to 69% and to 75% at 30th cycle, resulting in a net 6% reduction in the resistance change at 30th cycle with respect to the first cycle.

To identify the reason for the hysteresis and relaxation behavior, it is necessary to confirm the causes of change in resistance when the CPCs containing nanofillers are pressed. When a composite with a conductive filler is subjected to pressure, the pressure induces translation and deformation of the filler, resulting in a piezoresistive effect, and the pressure sensitivity is related to the mobility of the nanofiller [39,40]. There are two main reasons that explain the resistance change with pressure.

(1) Change in gap size between fillers: When pressure is applied, as the distance between adjacent fillers decreases, a new conductive path is formed, or electrical resistance in a current conductive path is reduced. Alternatively, by increasing the distance between the fillers through transverse slippage of the filler, the conductive path is destroyed.
(2) Intrinsic deformation of the filler by pressure: This is applicable in CNTs where curvature occurs with pressure, or a change in relative alignment takes place, resulting in the change in electrical resistance.

CNTs and CB have different geometric properties; therefore, the composites made from each of them show distinct tendencies on application of pressure. As CNTs are one-dimensional fillers, bending can occur when a composite made of CNTs is subjected to pressure [41]. However, because CB is a 0-dimensional filler, the fillers only translate when a composite made of CB is pressed [42]. Therefore, given the same force, the CNT/PDMS composites exert less pressure on the fillers to cause translation than the CB/PDMS composite. Therefore, more translation occurs in CB than in CNTs, which causes agglomeration, and thus, greater hysteresis could be observed. In addition, CB particles tend to aggregate into relatively weaker bonds than CNTs [28]. Thus, while being more aggregated, some electrical networks are repeatedly broken down and combine to cause deformation of the composite.

**Figure 6:**
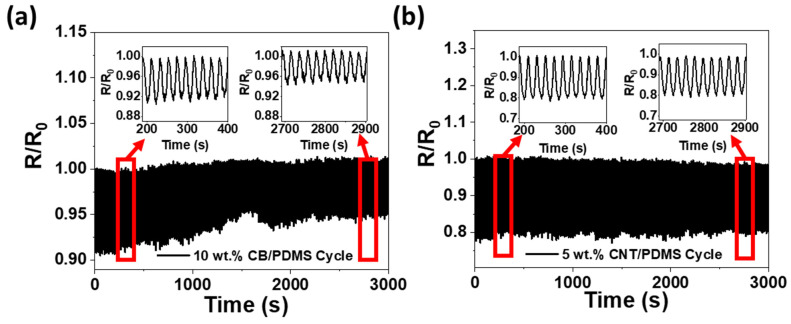
Normalized change in resistance (R/R_0_) under cyclic pressure deformation at 40 kPa for (**a**) 10 wt.% CB/PDMS and (**b**) 5 wt.% CNT/PDMS composites.

In order to verify the repeatability of the sensor through a successive pressure cycle according to the difference in fillers, a repetitive loading-unloading experiment was performed at 40 kPa after 30 cycles of relaxation. Figure 6 shows the relative resistance change in the loading-unloading cyclic experiment for the two CPCs for 3000 s. For 5 wt.% CNT/PDMS, stable repeatability was observed with no significant difference between the start and end of the cycle and stable resistance change for 3000 s, as shown in Figure 6b. However, in the case of CB/PDMS, unstable resistance changes were identified, and the resistance changes at the end of the cycle were reduced compared to those at the beginning of the cycle, as shown in Figure 6a.

**Figure 7:**
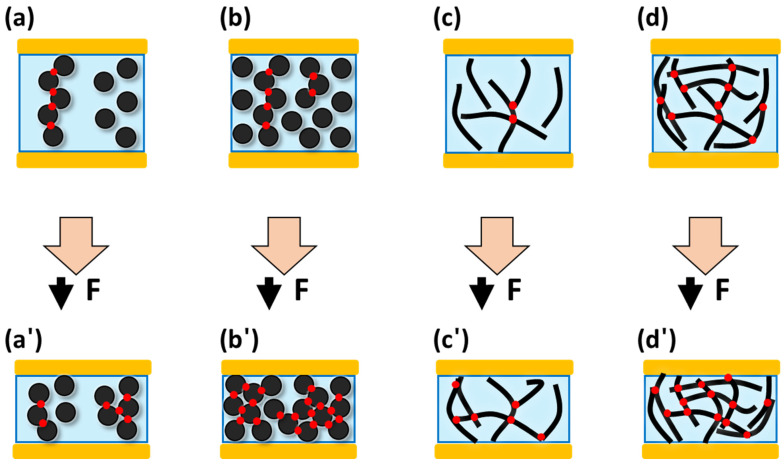
Scheme representing the effect of CB and CNT geometry on pressure sensing: (**a**,**a′**) low-content CB/PDMS composite, (**b**,**b′**) high-content CB/PDMS, (**c**,**c′**) low-content CNT/PDMS, and (**d**,**d′**) high-content CNT/PDMS (red: contact point, sky blue: polymer, yellow: electrode, black circle: carbon black, black line: CNTs).

Based on the results and discussions, a scheme (Figure 7) is proposed to explain the mechanism of the diverse pressure-sensing behaviors of CB/PDMS and CNT/PDMS. Figure 7a,a′ depict a low content CB/PDMS composite. It can be confirmed that an unstable conductive path exists before pressing, and the unstable electrical channel is destroyed when pressure is applied. Figure 7b,b′ show high content CB/PDMS composite. It is more likely to consist of conductive paths than low-content CB/PDMS. When pressure is applied, it can be observed that new conductive paths are formed even if the existing electrical network is destroyed. Figure 7c,c′ show low content CNT/PDMS composite. It can be observed that the one-dimensional materials, CNTs, are more likely to be in contact with each other than CB, irrespective of the low content. Figure 7d,d′ show the high-content CNT/PDMS composites. When the CNTs content is high, it can be seen that there are only a few new contact points formed when pressure is applied because sufficient electrical contacts already exist.

**Figure 8:**
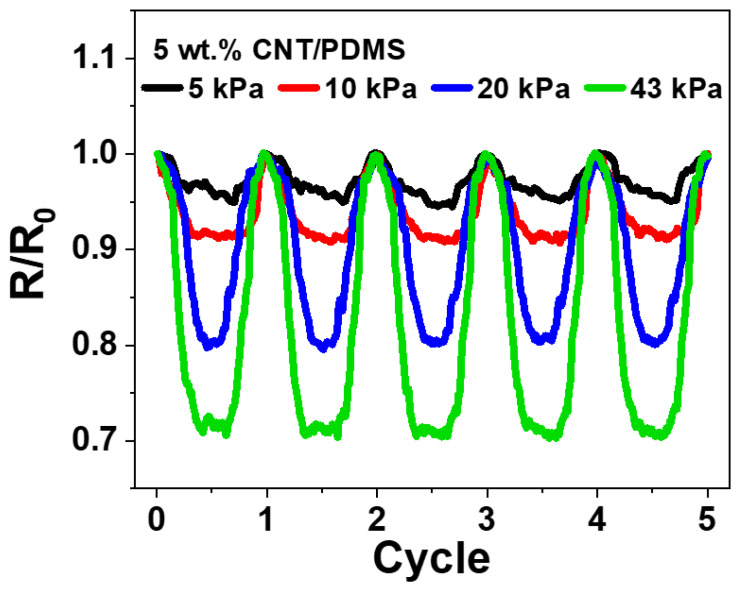
Normalized change in resistance (R/R_0_) of 5 wt.% CNT/PDMS for 5 cycles under varying pressure (5, 10, 20, 43 kPa).

As a result of all experiments, 5 wt.% CNT/PDMS displayed the lowest hysteresis and the highest pressing sensitivity. Hence, it was found to be most suitable for application as a pressure sensor. Experiments with successive loading and unloading of different forces were performed to analyze the pressure sensitivity of 5 wt.% CNT/PDMS. Figure 8 shows that the CNT/PDMS composite was subjected to five compression–release cycles at different pressures (5, 10, 25, and 43 kPa). Distinct curves can be observed for each pressure value which demonstrates potential application of the composite as a pressure sensor.

## 4. Conclusions

CB/PDMS and CNT/PDMS composites with different filler contents were prepared to compare their pressure-sensing properties according to different geometric structures. The percolation behavior of CPCs for each filler was measured. It was observed that the CNT/PDMS composite had a smaller percolation threshold than the CB/PDMS composite because CNTs have a larger aspect ratio than CB. A pressure test according to the content of each filler demonstrated that the higher the filler content, the higher is the sensitivity to pressure in the CPCs containing CB. In contrast, in CPCs containing CNTs, it was confirmed that the lower the filler content, the higher is the sensitivity to pressure. It has been suggested that the main reason for the different trends in the formation of electrical paths is the difference in the shapes of the fillers. According to the composition with the optimal pressure sensitivity for each filler, 5 wt.% CNT/PDMS and 10 wt.% CB/PDMS composites were selected for successive cycle experiments. CNT/PDMS was found to exhibit more stable hysteresis properties than CB/PDMS and it was confirmed that CPCs are stabilized through the pressure loading-unloading cycles. Further, CB/PDMS was observed to be more susceptible to pressure than CNT/PDMS during the stabilization process. Finally, the sensing performance of 5 wt.% CNT/PDMS was continuously measured at different pressures, which demonstrated great potential for application as a pressure sensor. In conclusion, CNTs are more advantageous than CB for fabricating pressure sensors with high piezoresistive effects, and repeatability and low hysteresis.

## Data Availability

Not applicable.
